# “Izervay (avacincaptad pegol): paving the way for vision preservation in geographic atrophy”

**DOI:** 10.1097/MS9.0000000000002021

**Published:** 2024-04-03

**Authors:** Laiba Shakeel, Afsheen Khan, Aymar Akilimali

**Affiliations:** a Department of Internal Medicine, Dow University of Health Sciences, Karachi, Pakistan; b Department of Research, Medical Research Circle (MedReC), Bukavu, Democratic Republic of Congo

**Keywords:** aptamers, complement C5, complement membrane attack complex, geographic atrophy, macular degeneration, nucleotide

## Abstract

Age-related macular degeneration (AMD) is a progressive retinal disease that primarily affects the macula, leading to central vision loss and impaired color vision. Among its most severe forms is geographic atrophy (GA), which results in irreversible central blindness. While numerous risk factors, including age, smoking, and genetics, contribute to the development of AMD, effective treatment options for GA have been limited. This article centers on Izervay [avacincaptad pegol (ACP)], an FDA-approved drug designed to address the unmet medical needs of patients with GA secondary to AMD. The pathophysiology of GA involves oxidative damage, chronic inflammation, and cell death, primarily due to complement system dysregulation. Previous treatments for GA have shown limited efficacy, leaving patients searching for more effective solutions. Izervay, with its unique mechanism of action, inhibits complement protein C5, disrupting the formation of the membrane attack complex and slowing retinal cell degeneration. Clinical trials have demonstrated Izervay’s ability to significantly reduce the growth of GA lesions, offering hope for improved outcomes. Additionally, the drug has exhibited a tolerable safety profile, with common side effects including conjunctival hemorrhage and increased intraocular pressure. Izervay represents a breakthrough in AMD treatment, offering the potential to preserve vision in those at risk of irreversible vision loss due to GA. While further research is necessary to evaluate long-term efficacy and accessibility, its approval opens new possibilities in AMD management, transforming the lives of individuals affected by this condition.

## Introduction

Age-related macular degeneration (AMD) is a progressive retinal disease primarily affecting the macula, responsible for visual acuity and color vision. Pathological changes in the deeper retinal layers and surrounding vasculature result in central vision loss. While age represents the most significant risk factor for AMD, others include smoking, elevated BMI, hypertension, hyperlipidemia, and heredity^1^. Around one million individuals experience geographic atrophy (GA), a severe form of dry AMD, with 160 000 new cases reported annually^2^. AMD has three phases: early, intermediate, and advanced, the latter being divided into dry AMD and wet (neovascular) AMD^3^. Atrophic lesions in the outer retina characterize GA, while wet AMD involves blood vessel leakage into the macula, leading to abrupt vision impairment. GA, unlike wet AMD, is a chronic condition that ultimately results in irreversible central blindness, with a median progression time to permanent blindness estimated at 6.2 years^4^.

This article is focused on Izervay, an FDA-approved drug for GA secondary to AMD. It provides an insight into its characteristics, mechanism of action, and potential role in managing GA secondary to AMD. It also explores other possible treatment options, enhancing the understanding of Izervay’s benefits in treating this disorder.

## Pathophysiology

**Figure 1:**
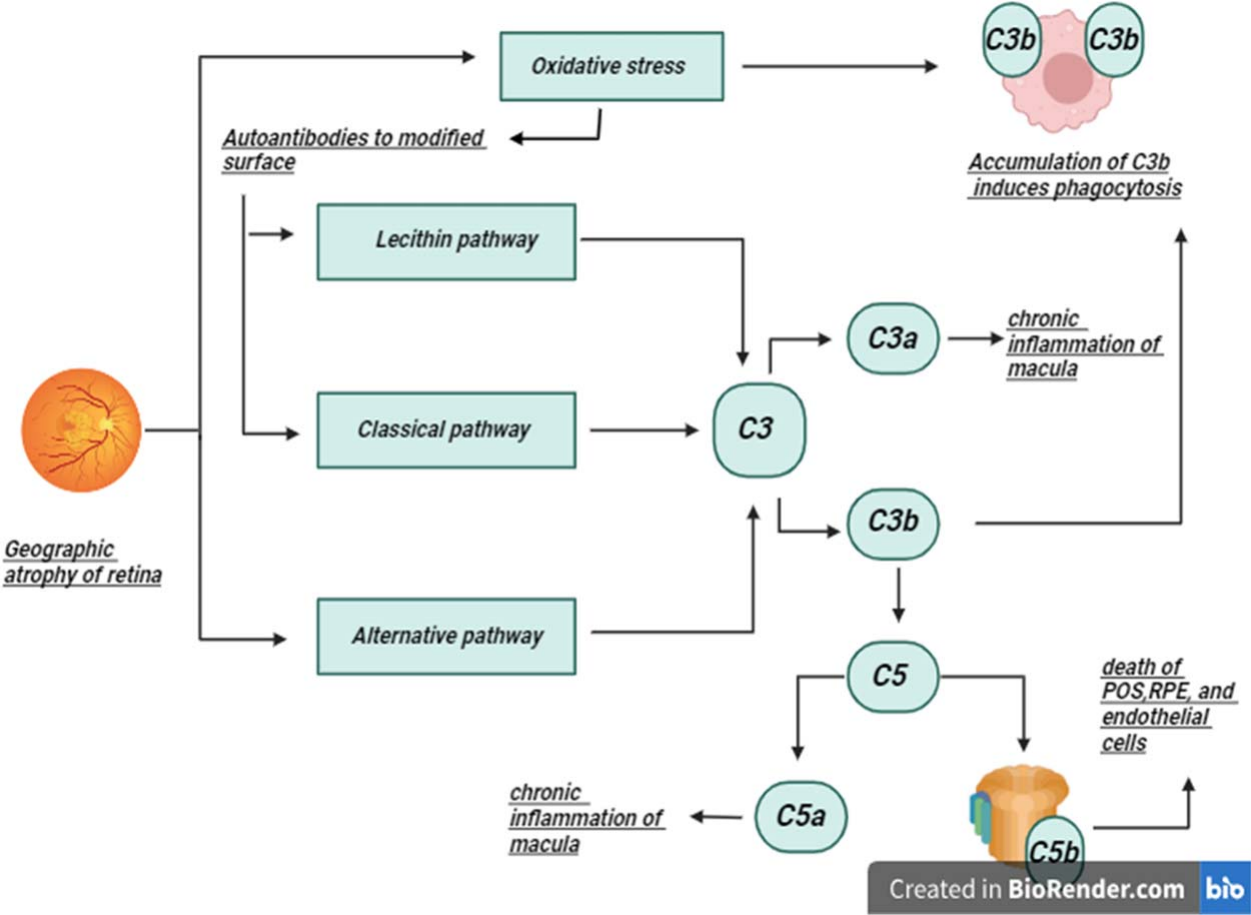
Pathophysiology of geographic atrophy.

GA secondary to AMD is a complex condition influenced by environmental stressors and aging factors. It involves accumulating oxidative damage, resulting in drusen deposits between the retinal pigment epithelium (RPE) and Bruch’s membrane. Excessive drusen accumulation triggers chronic inflammation through the complement cascade. This inflammation can lead to the death of crucial cells like photoreceptors, RPE, and choriocapillaris, causing sharply defined atrophic lesions and exposing choroidal vessels^4^. The complement system plays a significant role, with C3 and C5 fragments found in Drusen^4^ (Refer to Fig. 1). GA results in scotomas and visual impairments, even outside the fovea. It features atrophic lesions progressing from the outer retina towards the fovea, leading to irreversible vision loss^4,5^. Proper regulation of the complement system is essential, and genetic mutations in the regulatory complement protein are common in individuals with dry AMD^6^.

## Previous treatment

Treatment options for AMD secondary to GA have been explored with varying degrees of success. Unlike exudative AMD, which can be effectively managed with antivascular endothelial growth factor medications, available treatments for GA have limited proven efficacy. Patients have been recommended lifestyle changes, nutritional supplements, and vitamins, but their effectiveness in managing GA still needs to be proven^7^.

Several potential therapies have been investigated, including Brimonidine, a selective 2 adrenergic receptor agonist that has shown promise in providing neuroprotection by stimulating cell survival signaling and the production of brain-derived neurotrophic factor. However, it has also been associated with vascular injury and side effects like conjunctivitis and eye discomfort^8^.

NGM621, a humanized monoclonal antibody targeting complement C3, aimed to slow GA progression but fell short in the Phase 2 clinical trial, CATALINA, regarding its primary endpoint^9^. Gene therapies are promising to improve retinal disease treatment, with GT005, an investigational gene therapy seeking to enhance complement factor I (CFI) production, which can help restore complement system balance and reduce inflammation. Ongoing Phase 2 clinical trials, EXPLORE and HORIZON, further investigate the administration of GT005 in GA patients^10^.

Izervay, a novel treatment for GA, demonstrates promising efficacy compared to existing therapies. While Syfovre, the first FDA-approved GA treatment, exhibits a 36% slowdown in disease progression with monthly injections by targeting the immune system protein C3, it also raises concerns due to the rare but severe side effect of retinal vasculitis, which can lead to blindness by blocking retinal blood flow. In contrast, Izervay offers a potentially safer alternative, providing comparable efficacy without the same risk of severe adverse effects. Its effectiveness, safety profile, and overall outcomes suggest it is a valuable contender in GA treatments^11^.

## Izervay (avcincaptad pegol)

The FDA’s approval of Izervay [avacincaptad pegol (ACP)] represents a significant advancement in addressing the unmet medical needs of patients with GA secondary to AMD. ACP is an RNA aptamer, a PEGylated oligonucleotide, that plays a pivotal role in inhibiting complement protein C5. The primary action of ACP is the inhibition of C5, a component of the complement system. By binding to C5, this drug effectively prevents the cleavage of C5 into its biologically active fragments, C5a and C5b. This inhibition is significant because it disrupts the subsequent formation of the membrane attack complex (MAC). Inhibiting these C5-mediated has the potential to slow the progression of retinal cell degeneration in GA, offering therapeutic benefits. In patients with AMD, the growth of GA reflects the loss of photoreceptors and the underlying disease progression. Studies GATHER1 and GATHER2, which investigated the efficacy of ACP, revealed notable reductions in the rate of GA growth throughout treatment. This reduction in GA progression during the first year of ACP treatment demonstrates the drug’s potential to slow the advancement of GA secondary to AMD^12^.

Izervay’s unique mechanism of action in inhibiting complement protein C5 and preventing MAC formation represents a revolutionary breakthrough in treating GA associated with AMD.

## Clinical trial

The safety and efficacy of ACP, a C5 inhibitor, were assessed for GA secondary to AMD in a randomized, double-masked, sham-controlled clinical trial (As shown in the Table 1). In this randomized control trial, participants had to be at least 50 years old and have best-corrected visual acuity in the study eye that fell between 20/25 and 20/320 to be eligible. The GA had to be nonfoveal centered, secondary to AMD, and partially positioned within 1500 mm of the foveal center. In part 1 of the trial, 77 participants were randomized into three groups receiving monthly intravitreal injections of ACP at 1 mg, ACP at 2 mg, or a sham treatment. In the second half, 209 participants were randomized into three groups receiving monthly ACP at 2 mg, ACP at 4 mg, or a sham treatment. When compared to their respective sham groups, the effects of ACP treatment on GA growth were seen as early as month 6, with a 28.4% reduction for avacincaptad 2 mg and a 26.6% reduction for ACP 4 mg dose. When compared with their respective sham-control groups, the mean GA growth rate was seen to have decreased by 30.5 and 25.6%, respectively, after receiving monthly therapy with ACP 2 mg and 4 mg without undergoing square root transformation from baseline to month 12. ACP treatment was generally well-tolerated, and ocular adverse events were mainly related to the injection procedure. The study concluded that ACP at 2 and 4 mg demonstrated a continued reduction in GA progression and tolerability^13^. Similarly, A phase 3 trial, GATHER2, involved 448 participants from 205 retina clinics, research hospitals, and academic institutions worldwide. To qualify, patients need to have the best corrected visual acuity in the study eye between 20/25 and 20/320 and noncentrepoint involving GA to be 50 years of age or older aimed to evaluate the safety profile of ACP 2 mg in reducing GA lesion growth over 12 months. The patients were randomized in a 1:1 ratio, receiving ACP and sham. Of the patients in the ACP 2 mg group, 154 (68%) were female, and 71 (32%) were male; in the sham group, there were 156 (70%) female and 66 (30%) male patients. A significant difference in growth of 0·056 mm/year was observed between the ACP 2 mg group and the sham group from baseline to month 12. The trial found that ACP was well-tolerated and considerably slackened the growth of GA lesions compared to sham treatment. This suggests that ACP has the potential to alter the disease progression for patients with GA, offering hope for improved outcomes^14^.

**Table 1:** Summarised clinical trials showing the efficacy of izervay

Study ID	Phase	Sample size	Outcomes
GATHER-1 TRIAL (NCT02686658)^13^	Phase 2/3	Two hundred eighty-six participants with geographic atrophy, aged 50 or older	Treatment with Avacincaptad pegol showed a reduction in the mean rate of geographic atrophy growth over the 18 months
GATHER-2 TRIAL(NCT04435366)23	Phase 3	Four hundred forty-eight people with geographic atrophy secondary to AMD, aged 50 or older	Avacincaptad pegol was well-tolerated and significantly slowed the growth of geographic atrophy lesions compared to sham treatment

Reference:

Jaffe GJ, et al. 2021^13^.

Khanani, et al. 2023^14^.

## Recommend dosage and side effects

Izervay (ACP) carries specific potential side effects and precautions that should be carefully considered upon use. Mild side effects reported include eye pain, blurred vision, eye floaters, swelling or irritation around the eyelashes, and temporary red or bloody spots on the white part of the eye. Additionally, mild allergic reactions have been observed. More severe complications such as endophthalmitis (severe inflammation inside the eye), detached retina, increased risk of ‘wet’ AMD, and increases in eye pressure have also been reported. Before using Izervay, it is vital to confirm that the patients are not allergic to the active ingredient ACP or any other components in the medication. Izervay should not be used in ocular or periocular infections or active intraocular inflammation cases^15^. In addition to these adverse effects, Izervay costs around $2100 per injection. The high cost can affect its use as a sustainable treatment option and can hinder patient compliance with the entire treatment process^11^.

The recommended dosage for Izervay is a 2 mg intravitreal injection administered by a qualified physician once monthly for 12 months. It is imperative to store Izervay in the refrigerator within the temperature range of 2°C–8°C (36°F–46°F) and avoid freezing or shaking the vial^15^.

## Conclusion

In conclusion, Izervay (ACP) represents a significant milestone in the treatment of GA associated with AMD. By decelerating the progression of GA, it offers the hope of maintaining a better vision for an extended period. Administered via intravitreal injection, Izervay demonstrates promise as a vital intervention for those at risk of irreversible vision loss due to GA. As we move forward, it is crucial to conduct further research and clinical trials to assess its long-term efficacy and safety. Moreover, efforts should be made to increase its production, making it readily accessible worldwide and cost-effective to improve the quality of life for individuals affected by GA while alleviating the healthcare system’s burden. Izervay’s approval opens new possibilities in AMD treatment, enhancing the prospects of preserving vision, and ultimately transforming the lives of those afflicted by this condition.

## Ethical approval

Ethics approval was not required for this editorial.

## Consent

Informed consent was not required for this editorial.

## Sources of funding

The authors did not receive any financial support for this work. No funding has been received for the conduct of this study.

## Author contribution

L.S.: conceptualization and project administration; L.S. and A.K.: original draft of manuscript; A.A.: reviewing and editing the manuscript; A.K.: visualization.

## Conflicts of interest disclosures

Not applicable.

## Research registration unique identifying number (UIN)

Not applicable.

## Guarantor

Laiba Shakeel and Aymar Akilimali.

## Data availability statement

Not available.

## Provenance and peer review

Not commissioned, externally peer-reviewed.
